# Challenges in the Diagnosis and Management of Methamphetamine-Induced Intestinal Ischemia in a General Hospital With Limited Infrastructure: A Case Report

**DOI:** 10.7759/cureus.89733

**Published:** 2025-08-10

**Authors:** Adrian A Santos-Vega, Alejandra Y Proa-Arriaga, Fernando Martínez-Cuspinera, Sergio D Sánchez-González, Alejandra Carrera-Holguín

**Affiliations:** 1 General Surgery, Hospital Universitario de Saltillo, Saltillo, MEX; 2 General Surgery, Hospital General de Torreón, Torreón, MEX

**Keywords:** acute abdomen, colonic necrosis, intestinal ischemia, methamphetamine abuse, non-occlusive mesenteric ischemia, resource-limited hospital

## Abstract

Non-occlusive mesenteric ischemia (NOMI) is an abdominal emergency with high mortality, the confirmation of which usually relies on computed tomography, a resource unavailable in many second‑level hospitals in Mexico. We report a case of a 37‑year‑old man with no prior comorbidities and a four‑year history of chronic methamphetamine use who presented with diffuse abdominal pain of 10 days’ duration, fever, and incipient shock. Plain abdominal radiography demonstrated massive colonic distention. As computed tomography was unavailable and signs of peritoneal irritation were present, urgent exploratory laparotomy was performed, revealing patchy transmural necrosis of the entire colon with viable small bowel. Total colectomy and ileostomy were undertaken. Despite intensive vasopressor support, the patient died of refractory vasoplegic shock. This case underscores that NOMI secondary to methamphetamine use can occur in young patients and rapidly progress to massive colonic necrosis. It also highlights the need to maintain a high index of suspicion and proceed to early surgical exploration when the clinical scenario warrants it, even in settings with limited diagnostic resources, to avoid fatal delays.

## Introduction

Acute mesenteric ischemia (AMI) remains one of the most lethal abdominal emergencies, with mortality rates of 40-50% despite diagnostic and therapeutic advances [1]. Guidelines from the World Society of Emergency Surgery emphasize that prognosis improves with early recognition and prompt surgical or endovascular intervention; however, these recommendations rely on the availability of multidetector computed tomography (CT) and angiography, resources that are not always present in second‑level or rural centers [2]. Even in fully equipped hospitals, clinical presentation is often nonspecific, and diagnostic delay is common [3].

Within AMI, non-occlusive mesenteric ischemia (NOMI) accounts for up to 30% of cases and is caused by severe mesenteric vasospasm secondary to hemodynamic imbalance or sympathomimetic drugs [4]. Illicit substances such as methamphetamine trigger massive catecholamine release and prolonged splanchnic vasoconstriction, a mechanism implicated in "patchy" intestinal necrosis that may spare the small intestine and selectively compromise the colon [5]. Although the literature increasingly reports such cases, most originate from tertiary hospitals, leaving a knowledge gap regarding management in resource‑limited settings [6-8].

We present a case of a 37‑year‑old man treated in a general hospital in Mexico without CT access, whose clinical picture mandated urgent exploratory laparotomy that revealed massive colonic necrosis associated with chronic consumption of the drug colloquially known as "crystal." This report aims to highlight the need to recognize this emerging etiology to prevent lethal diagnostic delays.

## Case presentation

A 37‑year‑old male presented for his first visit to the emergency department on May 6th, 2025, reporting 10 days of progressively worsening abdominal pain. He had no relevant medical history, but he admitted to habitual methamphetamine use for the previous four years. The exact dosage was unavailable, but he stated that he had stopped using ''crystal'' when the pain began. The pain was insidious, generalized at evaluation, diffuse, rated 10/10 on a numeric rating scale, and accompanied by nausea, oral intolerance, absence of flatus, and a five-day history of profuse watery stools (Bristol type 7). On arrival, his vital signs revealed persistent tachycardia (144 bpm), blood pressure of 100/60 mmHg, and a fever of 38°C. Physical examination showed a distended, hyper-tympanitic abdomen with diminished bowel sounds and generalized guarding, consistent with peritoneal irritation.

**Figure 1:**
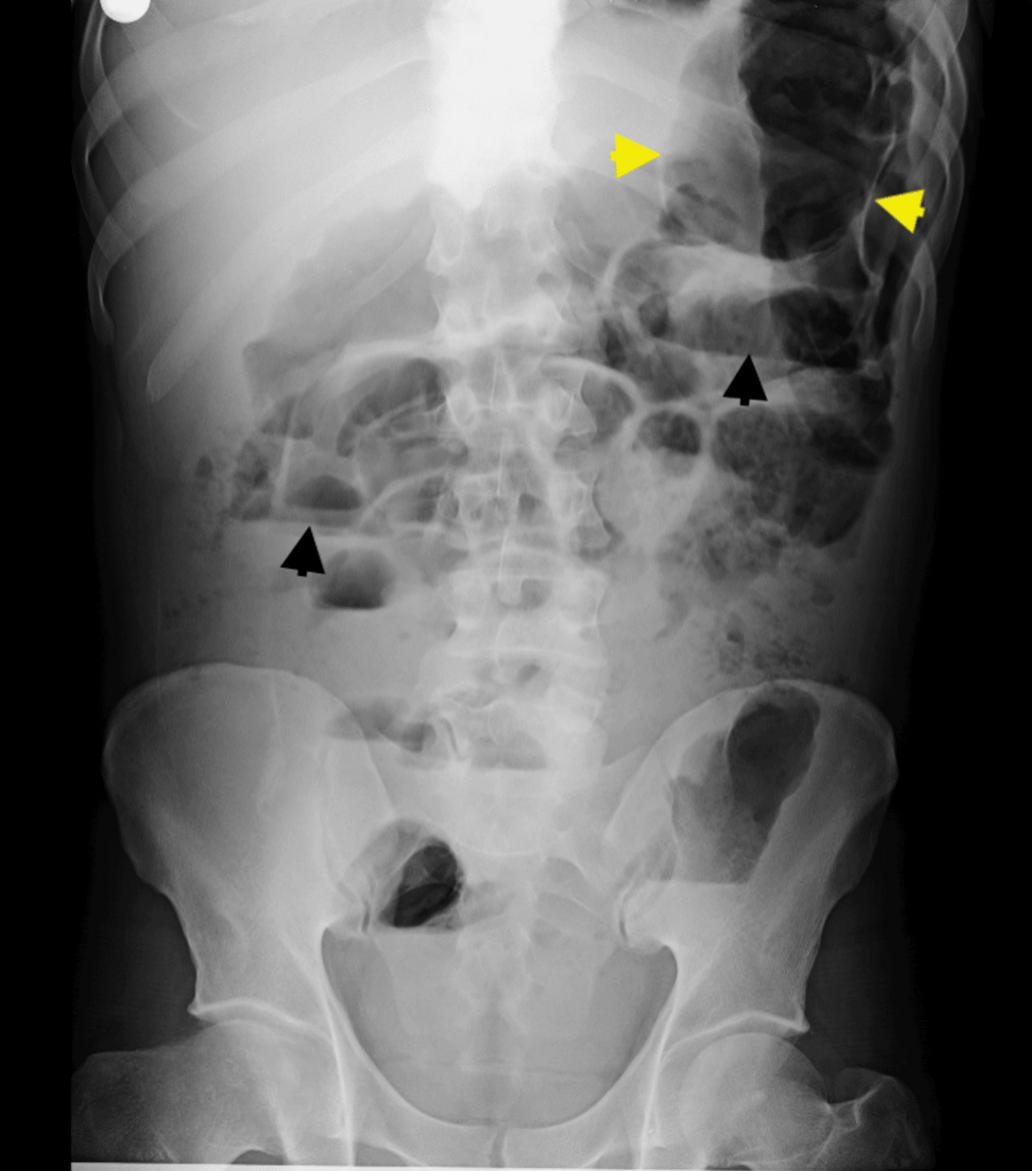
Upright abdominal radiograph. The black arrowheads mark the air-fluid levels, and the yellow arrowheads mark the distended bowel.

**Figure 2:**
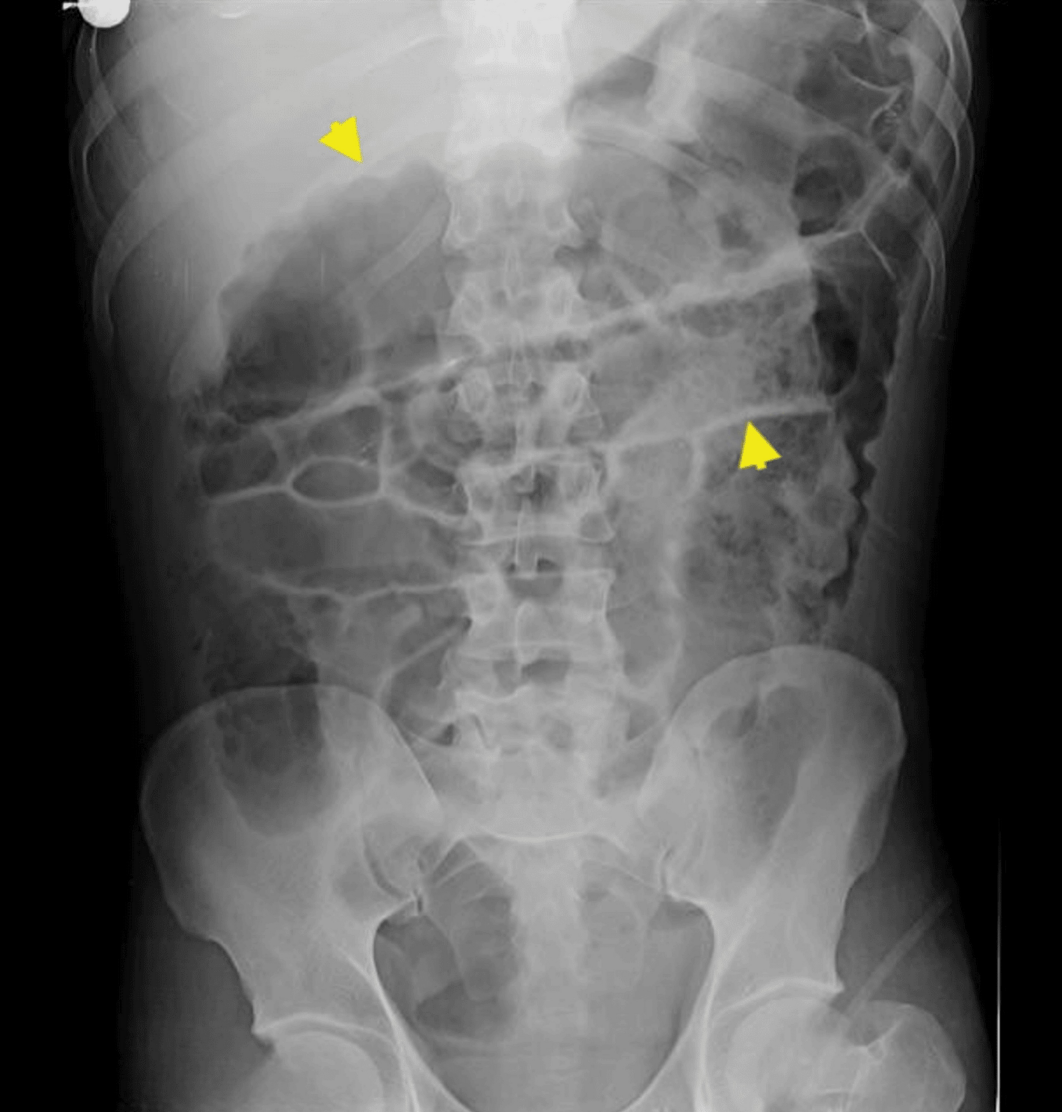
Supine abdominal radiograph. The yellow arrowheads mark the dilated bowel and the concomitant intestinal-wall thickening. Colonic loops measured 45 mm (ascending), 58 mm (transverse), and 56 mm (descending).

Upright and supine abdominal radiographs demonstrated multiple air-fluid levels and markedly distended bowel loops measuring 45 mm (ascending), 58 mm (transverse), and 56 mm (descending) without radiographic criteria for toxic megacolon (Figures 1, 2).

Initial laboratory studies taken on arrival showed normal hemoglobin and leukocyte count, with marked neutrophilia (87.9%), mild hyperglycemia (137 mg/dL), prolonged prothrombin time (15.9 seconds), and hypoalbuminemia (2.3 mg/dL). These findings reflected systemic stress, early hepatic hypoperfusion, and poor nutritional reserve, changes consistent with the splanchnic vasoconstriction and mucosal injury expected in NOMI. Relevant laboratory findings are presented in Table 1.

**Table 1:** Patient's laboratory findings. HIV: human immunodeficiency virus; VDRL: Venereal Disease Research Laboratory; PLR: platelet-lymphocyte ratio.

Test	Patient result	Reference range	Units
Hemoglobin	13.9	13.2 - 18.0	g/dL
Platelets	202 x 10^3^	150 - 420 x 10^3^	/μl
White blood count	5.8 x 10^3^	3.56 x10 - 10.30 x 10^3^	/μl
Neutrophil percentage	87.90%	39.6 - 76.1%	%
Absolute neutrophil count	5.1 x 10^3^	1.7 - 6.5 x 10^3^	/μl
Lymphocyte percentage	10.30%	15.5 - 48.6%	%
Absolute lymphocyte count	0.6 x 10^3^	1.0 - 4.0 x 10^3^	/μl
PLR	337	167.9 - 429.3	
Prothrombin time	15.9	9.9 - 12.9	seconds
Glucose	137	80 - 110	mg/dL
Creatinine	0.66	0.80 - 1.80	mg/dL
Total bilirubin	2.83	0.20 - 1.20	mg/dL
Direct bilirubin	2.18	0.00 - 0.50	mg/dL
Albumin	2.3	3.5 - 5.2	g/dL
Rapid HIV test	Positive (+)	Negative (-)	
Rapid VDRL test	Positive (+)	Negative (-)	

**Figure 3:**
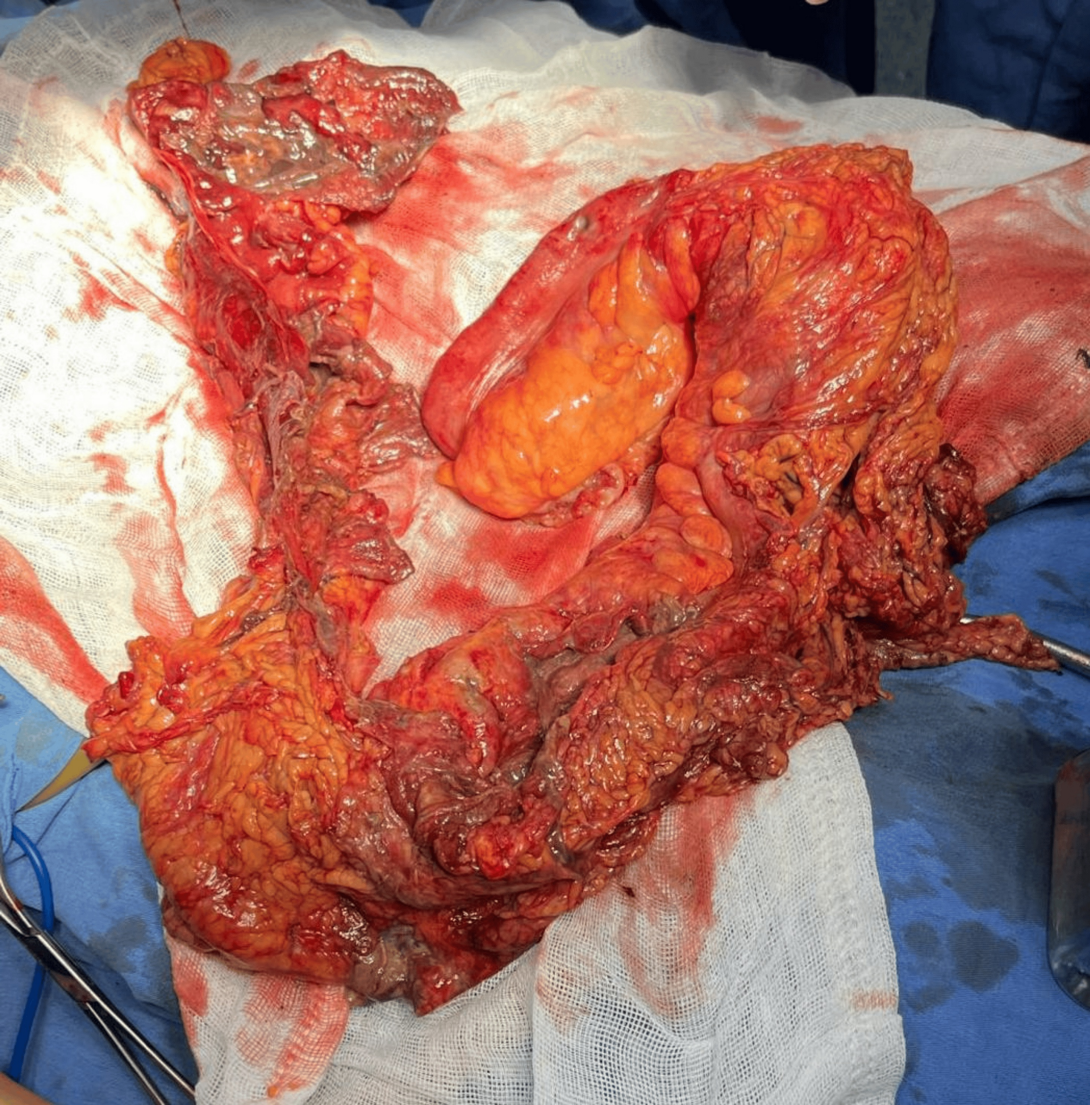
Gross specimen of total colectomy demonstrating diffuse colonic necrosis.

Due to limited resources, neither a drug panel nor further imaging was available. Given this pattern, plus the known vasospasm risk of stimulant abuse, the leading differentials were toxic megacolon, mechanical obstruction, and NOMI. Persistent tachycardia, peritoneal irritation, and diffuse colonic dilatation strengthened the suspicion of NOMI, so an urgent exploratory laparotomy was scheduled despite limited imaging resources. In the operating room, a central venous catheter was placed, and exploratory laparotomy revealed a fetid odor and "patchy" transmural necrosis throughout the colon with apparently viable small bowel. Total colectomy and ileostomy were performed, and a Penrose drain was left in the left paracolic gutter, the most affected area. Estimated blood loss was 800 mL (Figure 3).

The resected specimen was submitted for his histopathological examination, but the formal report could not be retrieved, and later attempts to locate it in the hospital records were unsuccessful, a recurrent and unfortunate consequence of logistics in a resource-limited setting.

Postoperatively, the patient remained hemodynamically unstable with tachycardia, tachypnea, and non‑perfusion mean arterial pressure, requiring high doses of norepinephrine. Ischemic changes were soon evident in the ileostomy, reinforcing suspicion of methamphetamine‑induced intestinal vascular compromise. He required intensive care, and transfer to a higher‑level facility was arranged; however, he died in the early hours of the same day.

## Discussion

Methamphetamine‑induced NOMI poses a particular diagnostic challenge in units with limited access to advanced imaging. Current guidelines recommend contrast‑enhanced CT as the first‑line study because it can reveal bowel wall thickening, pneumatosis, and absent enhancement; however, sensitivity decreases in early stages, and CT is not available in many second‑level hospitals [2,3,5]. In the patient, the absence of CT necessitated basing the surgical decision on the classic triad of severe abdominal pain, tachycardia, and peritoneal irritation, aligning with anecdotal reports in which early exploration was the only therapeutic opportunity [6-8].

Other recognized precipitants of NOMI can be systematically ruled out, such as vasopressor or digoxin exposure, thrombosis/embolism, fulminant infectious colitis, systemic vasculitis, and mesenteric arterial/venous thrombosis or embolism. These alternatives, the data that would have supported them, and the reasons we discounted them in this patient are summarized in Table 2.

**Table 2:** Differential causes considered for non-occlusive mesenteric ischemia. CMV: *Cytomegalovirus*; NOMI: non-occlusive mesenteric ischemia; HIV: human immunodeficiency virus; PAN: polyarteritis nodosa; SLE: systemic lupus erythematosus.

Potential cause	Supporting findings	Reasons ruled out in the patient
Vasopressor or digoxin therapy	Pharmacologic vasoconstriction can trigger NOMI	No vasoactive drugs prior to admission
Opportunistic/fulminant infectious colitis (*Clostridium difficile*, CMV in HIV)	Profuse diarrhea, HIV+	No marked leukocytosis, no radiologic megacolon, no recent antibiotics; necrosis was transmural rather than mucosal
Systemic vasculitis (PAN, SLE)	Can cause segmental intestinal ischemia	No rash, arthralgias, renal involvement; ischemia limited to the colon
Mesenteric arterial/venous thrombosis or embolism	Classically causes severe pain	Laparotomy revealed palpable mesenteric pulses and no thrombus; the small bowel was viable

Catecholamine‑mediated vasospasm explains the "patchy" colonic involvement observed, a finding described in experimental models and prior stimulant abuse case reports [4-6]. Conventional prognostic scales, such as Acute Physiology and Chronic Health Evaluation II (APACHE II), Sequential Organ Failure Assessment (SOFA), Simplified Acute Physiology Score II (SAPS II), show moderate utility for estimating mortality but require complete laboratory panels that may not be immediately available in peripheral hospitals [9]. Newer tools, such as multifactorial nomograms based on easily obtainable clinical and analytic variables, promise greater individualized accuracy [10]. Likewise, simple biomarkers, for example, a platelet‑to‑lymphocyte ratio > 204, have been correlated with 30‑day mortality and could be incorporated in basic‑resource settings [11].

Published cases reflect that, even after extensive resection, outcomes are poor due to vasoplegic shock and multiorgan failure [6-8]. Nevertheless, the interval from pain onset to intervention correlates inversely with survival, reinforcing the need to consider methamphetamine‑related NOMI in any young patient with disproportionate abdominal pain and stimulant use history, even when imaging studies are not immediately available.

## Conclusions

This experience shows that methamphetamine‑associated NOMI can occur in second‑level hospitals where CT access is limited and that diagnostic delay dramatically worsens prognosis.

Maintaining a high index of suspicion, recognizing that methamphetamine toxicity triggers fulminant mesenteric vasospasm, and proceeding to early surgical exploration when clinically indicated are critical steps to improve survival. Disseminating reports from resource‑constrained environments is essential to raise awareness and foster adapted algorithms that enable timely diagnosis and treatment.
